# Supplementary material for: Patterns of Intron Gain and Loss in Fungi
Source: PLoS Biol. 2004 Nov 30;2(12):e422. doi: 10.1371/journal.pbio.0020422 (PMC532390; doi:10.1371/journal.pbio.0020422)
Supplement: Table S1 — Also available at http://genes.mit.edu/NielsenEtAl/. (4.3 MB ZIP). [file pbio.0020422.st001.zip › NielsenEtAl/html/123.html]

AN7672.1.NCU02635.1.MG00171.1.FG06856.1


```
 CLUSTAL W (1.82) Multiple Sequence Alignments - Introns Inserted


Sequence 1: MG00171.1	373 aa
Sequence 2: FG06856.1	375 aa
Sequence 3: NCU02635.1	373 aa
Sequence 4: AN7672.1	379 aa
Alignment Length: 383 aa
Number Identitical Residues: 177 aa
Alignment Score (without introns) 9083


MG00171.1 	MARAMGPVRMKKANPITLAIGAFLCIFIIYFLAG-----PSGSSSNILKKKKAQPASHPL
NCU02635.1	MARPLGPVRLKRTSPGTIAVGIVLVIVILYLLVS-----PSAPDFSAAR--KATAALHPL
FG06856.1 	MARPMGAVRLKKTNPITLALGAILCIFIIVFLVS-----PSGKS-VTTRRFESVTAEHHL
AN7672.1  	MAVAR---KMRRTSPITIVLASLMAIGFLCFLFSSPSTPPASTSSTTTNSRLEDTAKHPL
          	** .    :::::.* *:.:. .: * :: :* .:.::.*:. .    .     .* * *

MG00171.1 	SPPTSPFRKESTSADGR-RTPPPVTRYNLNNVTTTANPIENREDVLILTPMARFYQGYWD
NCU02635.1	SPPTSPFRK-SSAVGGK-AGPPPVTRYNLNNVTITNDPVGNRENVLVLTPMARFYQEYWD
FG06856.1 	SPPTSPYRKSKSRSGVP-LKPPPVVHYNLNNVTTTTTPIENRENVLILTPMSRFYPGYWE
AN7672.1  	SPPTKPFIPQPVHGDGRHVQAPPVIKYDLNALTTSSDSTSHSERILILTPLTRFYQGYWD
          	****.*:  .    .     .*** :*:** :* :  .  : * :*:***::***  **:

MG00171.1 	NLLALNYPHELINLAFILPKNKEGNAATSALQAQVTKTQ---RGPKQFKSITILRQDFDA
NCU02635.1	NLLKLSYPHELITLGFILPKTKEGNAATIELQKQIAKIQKHGLEKERFKSIIILRQDFDP
FG06856.1 	NLLRLNYPHELITLGFILPKTKEGNQATAELQEFIHKTQKHGREADRFKSIIILRQDFEP
AN7672.1  	NIEKLSYPHEIISLGFIIPHTKYGDAALSALEKAIAKTQT-GPEENRFASITILRQDFDP
          	*:  *.****:*.*.**:*:.* *: *   *:  : * *. .   .:* ** ******:.

MG00171.1 	PLQSQDEAERHKMQNQKPRRAAMAKARNSLLFTTLGPSISWVLWLDGDIIETPPSLIQDL
NCU02635.1	PIPSQDESERHKMANQKARRAAMAKARNSLLFTTLGPDTSWVLWLDADIIETPPTLIQDL
FG06856.1 	AIISQDESERHKLKNQKARRAVMSKARNSLLFTTLGPDTSWVLWLDADIIETPPTLIQDL
AN7672.1  	PLITQNEKERHKLENQKARRESMSRARNSLLFTTLGPATSWVLWLDSDIVETPETLIQDL
          	.: :*:* ****: ***.**  *::************  *******.**:*** :*****

MG00171.1 	AKHDKAVIAPNCFQRYVDEKTGKSAERPYDYNNWQDSATAQELATKMGPDDILLEGYQEM
NCU02635.1	ASHSKPLTVPNCFQRFYNAEKKAMDERPYDFNNWQDSPTAQELASKMGKDDILLEGYADM
FG06856.1 	AFFDKPLIVPNCFQRYYDEEHKQMAERPYDFNSWQDSETAQALAAKMGPDDILLEGYAEM
AN7672.1  	TSHNRPVIVPNCFQRYYNTDTKSYDVRPYDFNSWIDSSTAQALGDTLKPDEILLEGYAEL
          	: ..:.: .******: : .      ****:*.* ** *** *. .:  *:****** ::

MG00171.1 	ATYRALMAYMTTEDNNL-DYEIPLDGVGGAALLVKADVHRDGAMFPPFAFYHLIETEGFA
NCU02635.1	ATYRALMAYMYEAGADI-HTEVPLDGVGGTALLVKAEVHRDGAMFPAFPFYHLIETEGFA
FG06856.1 	PTYRMLMAYLATEGGDR-NMILPLDGVGGTALLVKADVHRDGAMFPPFSFYHLIETEGFA
AN7672.1  	PTYRTLMAYQADTKNPRPKRVIDLDGVGGTALMVKAEVHRDGAMFPAFPFYHLVETEGFA
          	.*** ****        ..  : ******:**:***:*********.*.****:******

MG00171.1 	KMSKRLGFQPTGLPNYK0VYHYNE
NCU02635.1	KMAKRLGWQATGLPNYK0VYHYNE
FG06856.1 	KMAKRLGWQPWGLPNYK0IYHYNE
AN7672.1  	KMAKRLGYSVHGLPDYF0VYHYNE
          	**:****:.  ***:*  :*****
```
